# Suppression of LOX activity enhanced seed vigour and longevity of tobacco (*Nicotiana tabacum* L.) seeds during storage

**DOI:** 10.1093/conphys/coy047

**Published:** 2018-09-28

**Authors:** Zhan Li, Yue Gao, Cheng Lin, Ronghui Pan, Wenguang Ma, Yunye Zheng, Yajing Guan, Jin Hu

**Affiliations:** 1 Seed Science Center, Institute of Crop Science, College of Agriculture and Biotechnology, Zhejiang University, Hangzhou, China; 2 Yunnan Academy of Tobacco Agricultural Sciences, Yuxi, P.R. China; 3 Yuxi Zhongyan Tobacco Seed Company Ltd, Yuxi, P.R. China

**Keywords:** Artificial accelerated aging, LOX activity, natural aging, seed vigour and longevity, tobacco storage

## Abstract

The preservation of seed viability and quality in storage is an important trait both for commercial and germplasm usage. To better explore potential mechanisms of tobacco seed deterioration, seed packed in cloth bag (C) and vacuum bag (V) were stored under room temperature (RT) and low temperature (LT, 18°C), and sampled periodically for laboratory testing. Seed stored in low temperature with vacuum bag (LT/V) owned the highest seed vigour after 25 months of storage and in room temperature with cloth bag (RT/C) lost seed vigour and germination ability after 20-month storage. Meanwhile, seed in RT/C notably increased about 5-fold endogenous hydrogen peroxide (H_2_O_2_), 4-fold malondialdehyde (MDA) contents, 12-fold Lipoxygenases (LOX) activity and 2-fold the expression of *NtLOX3* comparing with LT/V at the end of 15-month storage. In addition, regression analysis indicated that LOX activity was strongly negatively correlated with seed vigour as the *R*^2^ value reached 0.970 in RT/C. Furthermore, caffeic acid and catechin, the inhibitors of LOX activity, were applied to tobacco seeds pre-treatment and followed with artificial accelerated aging. Seeds pretreated with inhibitors, especially caffeic acid, reduced LOX activity by 50%, MDA and H_2_O_2_ contents by 40% and 20%, respectively, and increased more than 1.2-fold seed vigour and seedling quality comparing with seeds pretreated with H_2_O after 6-day artificial aging, indicating a better seed storability after artificial accelerated aging. These results suggest that LOX accelerated seed aging, and suppression of LOX activity enhanced seed vigour and viability in accelerated aging tobacco seed, opening new opportunities for effective management of seed germplasm under long-term storage and conservation.

## Introduction

Seed longevity, which is accompanied with a progressive loss of quality or viability over time, is a crucial issue for germplasm conservation and seed marketing (Agacka-Moldoch *et al.*, 2015). For germplasm conservation, the maintenance of *ex situ* seed viability over long periods of time in seedbanks is a key element (Fu *et al.*, 2015). Methods including storage under low seed moisture content and temperature or place with various packing, have been developed and applied to avoid the effects of external environment on seed viability (Martins *et al.*, 2004; Hopkinson and English, 2005; Van Treuren *et al.*, 2013; FAO, 2014). Tobacco seeds (*Nicotiana tabacum* L.) possessed high economic values and are the foundation of *N. tabacum* industries. Nevertheless, there has been no systematic and scientific researches of the relationship between external conditions and seed vigour or viability during tobacco seed storage.

Seed aging, in part, still followed the ‘free radical theory’ which posited that damage caused by the accumulation of free radicals was the underlying mechanism in the organism aging (Harman, 1993; Kibinza *et al.*, 2011). Numerous studies reported the importance of reactive oxygen species (ROS) in seed aging (Kibinza *et al.*, 2006; Chen *et al.*, 2013; Ahmed *et al.*, 2016). Hydrogen peroxide (H_2_O_2_), one of ROS, at proper concentration was conducive to seed dormancy-broken and germination enhancement, however, the over-accumulation of H_2_O_2_ easily caused cell injury (Kumar *et al.*, 2015). High concentration H_2_O_2_ generated as by-product of respiration metabolic processes (Laloi *et al.*, 2004; Giorgio *et al.*, 2007), was considered as the critical factor in contributing to seed deterioration and influencing seed longevity and vigour (He *et al.*, 2015; Debeaujon *et al.*, 2000; Murthy *et al.*, 2003). In addition, malondialdehyde (MDA), end-products of lipid peroxidation, caused cell damage by reacting with macromolecules (Sriyong, 2007; Zhan *et al.*, 2014). Lipid oxidation in lipid-rich seeds could be a predominant damaging process in aging seeds (Stewart and Bewley, 1980). It should be noted that the lipid content of the tobacco seeds can be up to 40% by weight (Giannelos *et al.*, 2002). Thus, avoiding damage induced by serious lipid peroxidation is momentously important to tobacco seeds during storage.

To achieve homoeostasis of H_2_O_2_ generation, seed evolved highly efficient repair systems, such as enzymatic antioxidant systems (Xia *et al.*, 2015). Catalase (CAT, EC 1.11.1.6) and ascorbate peroxidase (APX, EC 1.11.1.11) were responsible for scavenging over-accumulated H_2_O_2_ and maintaining ROS dynamic balance in leaves and seeds (Gajewska and Skłodowska, 2007; Li *et al.*, 2017). Meanwhile, lipoxygenases (LOX, EC1.13.11.12) played an important role during lipid peroxidation in rice (*Oryza sativa* L.), soybean (*Glycine max* (L.) Merr.), sweet lupin (*Lupinus* L.) and canola (*Brassica napus*) seeds (Xu *et al.*, 2015; Lima *et al.*, 2010; Stephany *et al.*, 2015; Terp *et al.*, 2006), which catalysed the oxygenation of polyunsaturated fatty acids, such as linoleic and linolenicacids, to form conjugated diene hydroperoxides (Axelrod *et al.*, 1981). In addition, the activation of enzymes and various metabolisms in seed required the participation of moisture, thus, the changes of moisture content was also vital for seed vigour during storage, which had been extensively studied on soybean, rice, *Lallemantia royleana* and oat seeds (Ajala *et al.*, 2012; Wang, 2014; Baladi and Balouchi, 2016; Kong *et al.*, 2014). Furthermore, the activity of LOX could be effectively inhibited by some phenolic compounds, where the most effective inhibitor was caffeic acid (about 57% of inhibition) (Szymanowska *et al.*, 2009), followed by catechin, a kind of flavonoids, considerably inhibited LOX activity in horse bean (*Vicia faba* L.) and barley (*Hordeum vulgare* L., Redrejo-Rodriguez *et al.*, 2004; Szymanowska *et al.*, 2009). The involvements of LOX in tobacco seed vigour remain unclearly.

Therefore, the present investigation was conducted to comprehend the deterioration process of tobacco seeds and validate a protocol for delaying seed aging and prolonging seed longevity. Seed vigour, seedling quality, antioxidant enzymes and LOX activities, and corresponding gene expression were determined after aging, to acquire a better understanding on potential mechanisms of seed deterioration and support the perspectives of LOX activity as a new sensitive signal for predicting seed aging under storage.

## Materials and methods

### Natural aging and seed moisture content

Two commercial tobacco seeds, Honghua Dajinyuan (HD) and Yuanyan97 (Y97), with the initial moisture content of 4.2% (on the dry weight basis) were obtained from Yunnan Academy of Tobacco Agricultural Sciences. Seeds packed in vacuum (V) and cloth (C) bag were stored for totally 25 months (from June 2015 to June 2017) under low (LT, 18°C) and room temperature (RT), respectively (LT/V, LT/C, RT/V, RT/C). RT referred to the temperature of Yuxi city, Yunnan Province (102.52E, 24.35N), China (Supplementary Fig. S11). And the temperature changed continuously from 22 to 7°C, and then rose to 22°C again in a year’s cycle. Seeds were sampled every 5 months for moisture content and other index determination. Seed moisture content measured according to high-temperature drying method (ISTA, 2013). Approximately 0.200 g of seeds were placed in a sample container and weighed, and then oven-dried at ~130–133°C for 1 h. After cooling for 30 min in a desiccator, seeds were weighed again and the moisture content [(initial weight minus dry weight)/initial weight] was calculated.

### Seed germination and seedling growth

The seed quality at different storage stages was evaluated by a standard germination test (ISTA, 2013). Four 100-seed replications for each stage were used, and seeds were germinated on three layers of water-saturated filter papers in diameter 10-cm germination dishes at 25°C, with a photosynthetic active photon flux density of 250 μmol·m^−2^·2^−1^ and a photoperiod of 8 h light (L):16 h dark (D). Based on the daily number of germinated seeds (radicle emergence), the germination index (GI) was measured according to GI = Σ (Gt/Tt), where Gt is the number of the new germinated seeds on day *t* and Tt is the time corresponding to Gt in days. Then the germination energy (GE) and germination percentage (GP) was calculated on the 7th and 16th days, respectively. After germination for 16 days, seedling length (SL) was manually measured on twenty randomly selected normal seedlings with a ruler, the dry weight of 50 seedlings (DW) was determined after drying at 80°C for 24 h, and vigour index (VI = GI×DW) was also calculated.

### The changes of enzymes, various metabolites and gene expression during seed aging

To investigate the cell damage or seed deterioration after aging, MDA and H_2_O_2_ content were measured firstly. The H_2_O_2_ content was determined with 0.2 g of seeds according to the method of Doulis *et al.* (1997), and calculated as μmol H_2_O_2_ decomposition min^−1^·g^−1^·FW.

MDA content was qualified by the thiobarbituric acid reaction method as described by Gao *et al.* (2009). Then, the antioxidant enzymes and Lipoxygenase (LOX) activities which were involved in seed repair systems were determined. About 0.1 g of seedlings per replication and four replications for each treatment were used to obtain enzyme crude extract with 0.1 mM potassium phosphate buffer (pH 7.8). The supernatant was stored at 4°C for enzyme activity assays. The activities of CAT and APX were determined at 25°C through the methods described by Qiu *et al.* (2005), and calculated as μmol ascorbate decomposition min^−1^·g^−1^·FW using a UV spectrophotometer (UV-2450, Shimadzu, Japan). For the LOX assays, linoleic acid substrate solution (10 mM linoleic acid) and sodium phosphate reaction buffer (150 mM, pH 8.0) were prepared as described previously (Stephany *et al.*, 2015). LOX reaction of 0.1 g seeds was measured by UV spectrophotometer at 234 nm.

In order to further explore the changes of enzyme activity at the molecular level, the gene expressions were subsequently detected. Total RNA was isolated from seeds using Trizol reagent (Huayueyang, Beijing, China) and reverse transcribed using a Rever Tra Ace qPCR RT kit (Toyobo, Osaka, Japan) following the manufacturer’s instructions. The RT-qPCR of *NtCAT3, NtAPX2, NtLOX3* was performed using Roche real-time PCR detection system (Roche life science, USA). Gene specific RT-PCR primers were designed based on their cDNA sequences (Supplementary Table S1). Each reaction (20 μL) consisted of 10 μL of SYBR Green PCR Master Mix (Takara, Chiga, Japan), 1 μL of diluted cDNA and 0.1 μM forward and reserve primers. The PCR cycling conditions were as follows: 95°C for 3 min, followed by 40 cycles of 95°C for 10 s and 58°C for 45 s. The tobacco *Actin* gene was used as an internal control. Relative gene expression was calculated according to Livak and Schmittgen (2001).

### Artificial accelerated aging verification

The regression analysis between seed vigour and physiological traits (enzymes and metabolites) in HD and Y97 seeds during natural aging was conducted. And the indicator which was most relevant with seed vigour was selected as the aim of following artificial accelerated aging verification. Caffeic acid (CF) and catechin (CT) were used as the inhibitors. Tobacco seeds were pretreated with H_2_O (H), 1mM CF and 1mM CT, respectively, for 12 h. Then, all pretreated seeds were air-dried at 25°C for 48 h to their original moisture contents, subsequently followed by artificial accelerated aging under high temperature (43°C) and high relative humidity (75%) for 0, 3 and 6 days. At each sampling stage, all the parameters mentioned above, such as seed germination, seedling quality, enzymes, metabolites and gene expression were also measured.

### Data analysis

Data were analysed by analysis of variance (ANOVA and MANOVA) using the Statistical Analysis System (SAS) (version 9.2) followed by calculation of the Least Significant Difference (LSD, *α* = 0.05). Percentage data were arc-sin-transformed prior to analysis. The regression analysis was evaluated by the Pearson correlation coefficient.

## Results

### Various changes during natural aging

#### Moisture content

**Figure 1:**
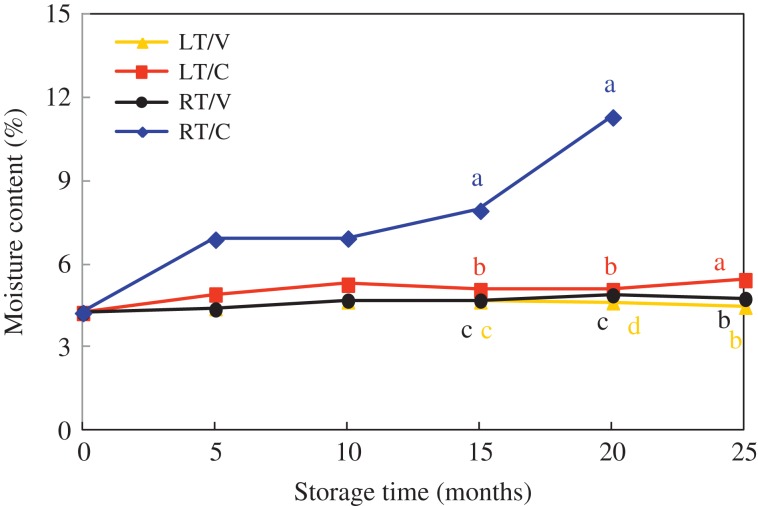
Dynamic changes in moisture content of HD seeds during natural aging. HD: Honghua Dajinyuan; LT/V: low temperature (18°C) with vacuum bag packing; LT/C: low temperature (18°C) with cloth bag packing; RT/V, room temperature with vacuum bag packing; RT/C: room temperature with cloth bag packing. Vertical bars above mean indicated standard error of two replicates of 0.2 g seeds each treatment. Different small letter (s) following the values indicated significant difference (LSD, *α* = 0.05) among treatments. Seed storage in RT/C lost germination ability after 20 months, parameters data were no longer concerned.

For HD seeds, moisture content in LT/V, LT/C and RT/V treatments basically remained at 4–5% in general during seed storage, which in RT/C rapidly increased from 4.3% to 11.3% during storage, especially from 15 to 20 months (Fig. 1). The same pattern could also be found in Y97 seeds (Supplementary Fig. S1), and moisture content in RT/C varied from 4.3% to 13.9% during storage (0–20 months). In addition, the interaction between storage time and storage pattern (packing and temperature) in HD and Y97 seed had significant effects on moisture content in HD and Y97 seeds as the *P*-value was 0.0001 (Table 3), suggesting the change in moisture content is determined by storage time and storage pattern together, not individual.

#### Seed germination and seedling quality

**Figure 2:**
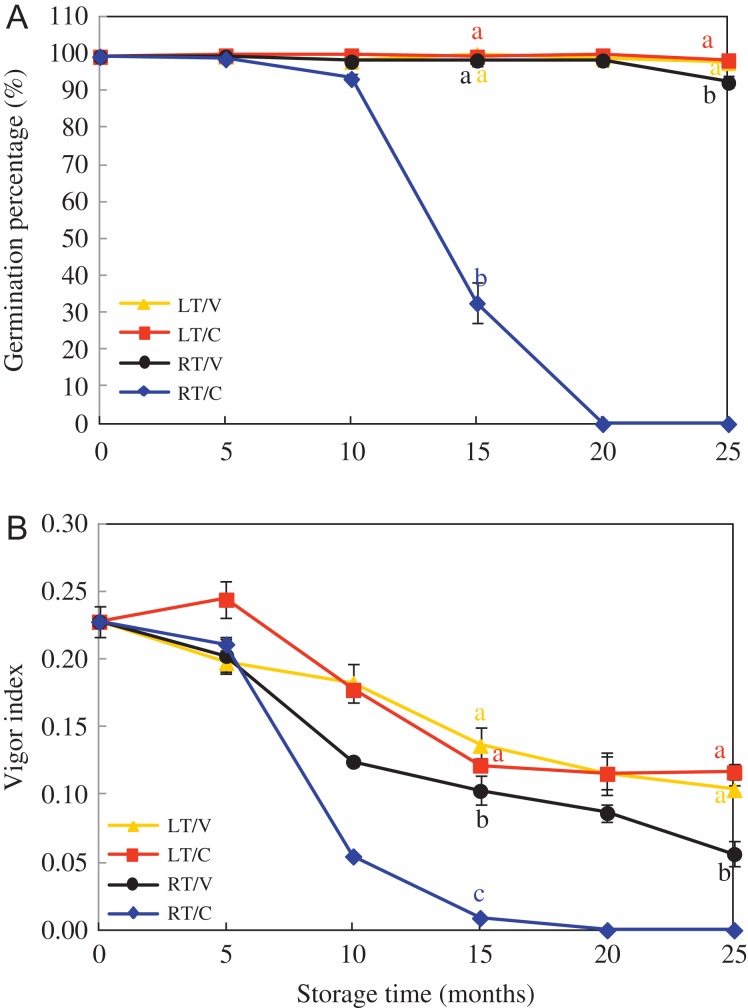
Seed viability and vigour of HD decreased as storage continued. Seeds were collected, respectively, in 0, 5, 15, 20 and 25 months after storage. HD: Honghua Dajinyuan. Germination percentage was calculated on the 16th day of germination test with four replications for each treatment. Vigour index was calculated using the formula: VI = GI×DW, where Gt is the number of new germinated seeds in time Tt, DW is dry weight. Different small letter (s) on the top of the bars indicated significant differences (LSD, *α*= 0.05) among treatments at same storage time. Error bars indicated ± SE of mean (*n* = 4).

GP and VI decreased in all treatments during storage (Fig. 2 and Supplementary Fig. S2). VI was about 0.22 in HD and Y97 seeds before aging, which reduced to about 0.10 in LT/V and LT/C, 0.06 in RT/V after 25 months of storage and <0.01 in RT/C after 15 months. The interaction between storage time and storage pattern (packing and temperature) in HD and Y97 seeds on VI was significant (Table 3). Moreover, GP in RT/C sharply decreased and dropped to 30.0% after 15 months, and completely lost viability after 20 months. Germination performance and seedling quality were paid main attention to storage of 15 (significant time node) and 25 months (end of storage).

After 15-month storage in HD and Y97 seeds, GE in RT/C was 0.0%, while in LT/V, LT/C and RT/V were all above 94% (Table 1 and Supplementary Table S2). As compared with CK (seeds before storage), GI and MGT in LT/V, LT/C and RT/V significantly declined and extended, respectively, while GI in RT/C was the lowest, and MGT was the longest. At the end of storage (25 months), the highest values of GI and DW were all recorded in LT/V.

**Table 1:** Seed vigour and seedling quality of HD seeds in response to different storage conditions during natural aging

	Treatments	GE (%)	GI	MGT (d)	SL (cm)	DW(g/50 plants)
	CK*	99.3 ± 1.1a/a	30.2 ± 0.1a/a	3.36 ± 0.05d/c	1.65 ± 0.37a/a	0.0076 ± 0.0004a/a
5	LT/V	99.3 ± 0.6	30.9 ± 0.6	3.27 ± 0.05	1.90 ± 0.07	0.0064 ± 0.0002
	LT/C	99.7 ± 0.6	31.8 ± 0.3	3.19 ± 0.06	1.86 ± 0.08	0.0077 ± 0.0004
	RT/V	99.3 ± 1.2	31.6 ± 0.3	3.19 ± 0.03	1.97 ± 0.05	0.0064 ± 0.0004
	RT/C	98.7 ± 1.2	31.1 ± 0.2	3.22 ± 0.02	1.97 ± 0.18	0.0068 ± 0.0001
10	LT/V	98.0 ± 0.0	30.8 ± 0.2	3.23 ± 0.02	1.87 ± 0.15	0.0059 ± 0.0005
	LT/C	99.7 ± 0.6	32.1 ± 0.2	3.13 ± 0.01	1.73 ± 0.56	0.0058 ± 0.0004
	RT/V	98.0 ± 2.6	26.2 ± 0.9	3.80 ± 0.06	1.73 ± 0.15	0.0048 ± 0.0002
	RT/C	20.3 ± 1.5	11.6 ± 0.4	8.09 ± 0.11	1.57 ± 0.12	0.0047 ± 0.0002
15	LT/V	100.0 ± 0.0a	27.7 ± 0.5b	3.68 ± 0.06c	1.75 ± 0.06a	0.0049 ± 0.0006b
	LT/C	99.3 ± 0.6a	27.9 ± 0.8b	3.63 ± 0.09c	1.74 ± 0.04a	0.0041 ± 0.0004c
	RT/V	98.3 ± 1.5a	25.4 ± 0.4c	3.91 ± 0.00b	1.25 ± 0.04b	0.0041 ± 0.0004c
	RT/C	0.0	2.0 ± 0.5d	9.44 ± 0.08a	1.30 ± 0.03b	0.0039 ± 0.0002c
20	LT/V	99.0 ± 1.0	26.8 ± 0.3	3.75 ± 0.02	1.76 ± 0.02	0.0043 ± 0.0004
	LT/C	99.7 ± 0.6	27.3 ± 0.2	3.71 ± 0.02	1.81 ± 0.05	0.0042 ± 0.0006
	RT/V	98.3 ± 1.5	21.5 ± 0.5	4.67 ± 0.06	1.27 ± 0.09	0.0040 ± 0.0002
	RT/C	0.0	0.0			
25	LT/V	97.7 ± 1.5a	26.8 ± 1.3b	3.71 ± 0.11b	1.66 ± 0.05a	0.0039 ± 0.0002b
	LT/C	98.3 ± 1.2a	26.6 ± 0.5b	3.76 ± 0.03b	1.57 ± 0.04a	0.0038 ± 0.0011b
	RT/V	92.3 ± 1.5b	19.3 ± 0.7a	4.86 ± 0.12a	1.17 ± 0.05b	0.0029 ± 0.0005b
	RT/C	0.0	0.0			

*Values were mean ± SE (*n* = 4). Different small letter (s) following the values indicated significant difference (LSD, *α* = 0.05) among treatments. Seed samples were collected, respectively, in 0, 5, 10, 15, 20 and 25 months after storage. The letters combination such as a/a in the row of CK, indicated the difference (LSD, *α*= 0.05) between CK and treatments after 15 and 25 months of storage, respectively. HD: Honghua Dajinyuan; GE: germination energy, calculated on day 7; GI: germination index, was measured as GI = ∑ (Gt/Tt); MGT: mean germination time, was calculated as MGT = ∑ (Gt × Tt)/∑Gt, where Gt is the number of new germinated seeds in time Tt; SL: seedling length; DW: seedling dry weight, was weighed directly after drying at 80°C for 24 h. LT/V: low temperature (18°C) with vacuum bag packing; LT/C: low temperature (18°C) with cloth bag packing; RT/V, room temperature with vacuum bag packing; RT/C: room temperature with cloth bag packing. Seed storage in RT/C lost germination ability after 20 months, parameters data were no longer concerned.

#### Endogenous H_2_O_2_ and MDA contents

**Figure 3:**
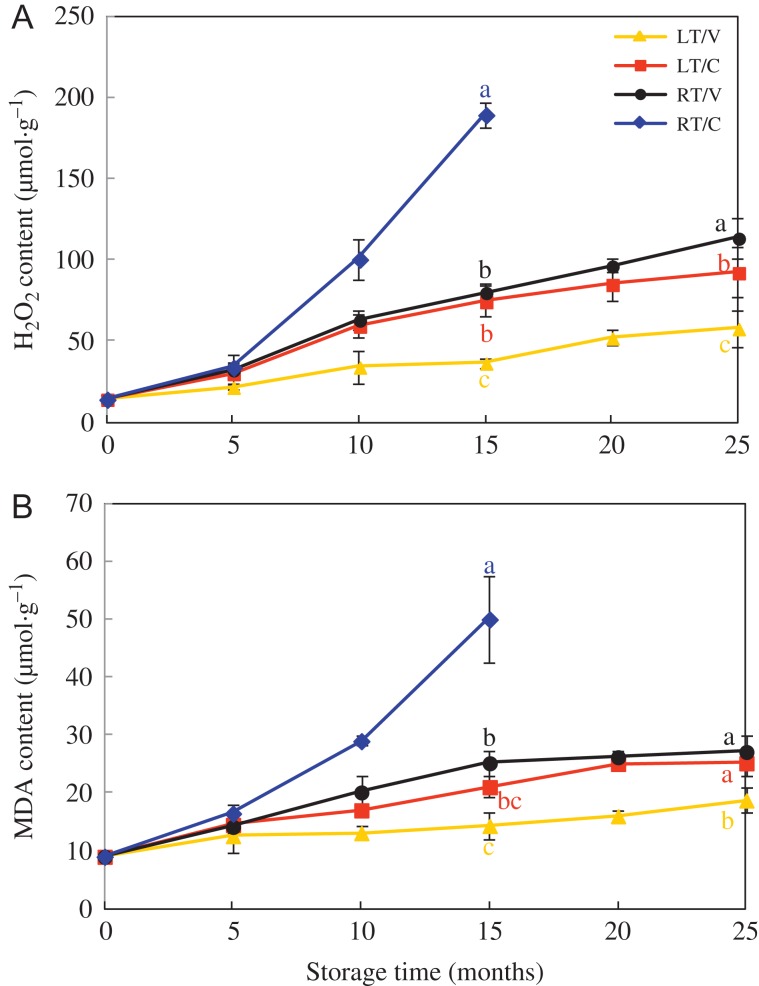
Seed storage increased endogenous H_2_O_2_ (**A**) and MDA (**B**) content in HD seeds. Seeds were collected, respectively, in 0, 5, 10, 15, 20 and 25 months after natural storage, and four replications for each treatment at each sampling time were used. HD: Honghua Dajinyuan; H_2_O_2_: hydrogen peroxide; MDA: malondialdehyde. Different small letter (s) on the top of the bars indicated significant differences (LSD, *α* = 0.05) among treatments at the same storage time. Error bars indicated ± SE of mean (*n* = 4). For additional explanations, see Fig. 1.

H_2_O_2_ content in HD and Y97 seeds gradually increased in LT/V, LT/C and RT/V, LT/V exhibited the lowest values, followed by LV/C and RT/V. While in RT/C, it increased sharply and was significantly higher than other treatments from 10 to 15 months of storage (Fig. 3A and Supplementary Fig. S3A). In addition, H_2_O_2_ content in RT/C reached about 5-fold higher than that in LT/V when storage for 15 months.

The changes of MDA content were similar to that of H_2_O_2_ in both HD and Y97 seeds (Fig. 3B and Supplementary S3B). MDA content in RT/C reached about 5-fold higher than that in LT/V after 15-month storage. At the end of storage, LT/V owned the lowest level of MDA content.

#### Antioxidant enzymes and LOX activities, and relative gene expression

**Figure 4:**
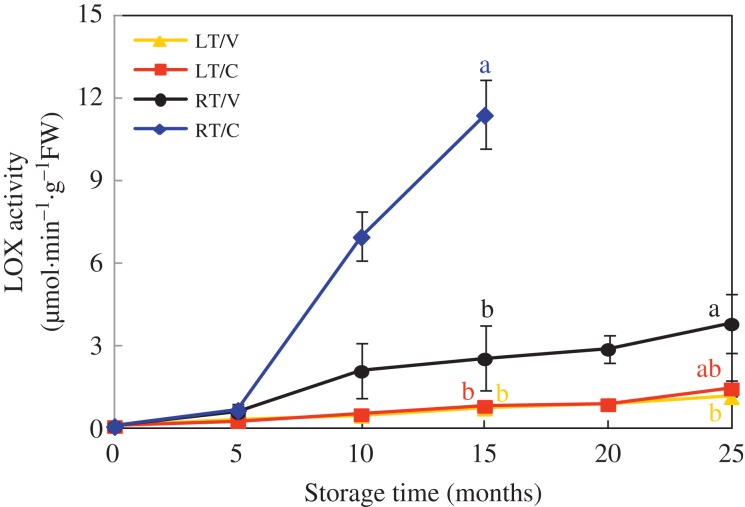
LOX activity increased accompanied by natural aging in HD seeds. HD: Honghua Dajinyuan; LOX, lipoxygenases. Seed samples were collected, respectively, in 0, 5, 10, 15, 20 and 25 months after storage, and four replications for each treatment at each sampling time were used. Different small letter (s) on the top of the bars indicated significant differences (LSD, *α* = 0.05) among treatments at the same storage time. Error bars indicated ± SE of mean (*n* = 4). For additional explanations, see Fig. 1.

CAT and APX activities of four treatments in HD and Y97 seeds fluctuated in the small-scale during storage showed in Supplementary Fig S4. After storage for 15 months, CAT activity of RT/C was lower than other three treatments. For APX activity, there were no significant difference among four treatments after 25-month storage. LOX activity showed a stepped upward tendency during storage and reached the highest level in 25 months except for RT/C (15 months) in HD and Y97 seeds (Fig. 4 and Supplementary S5). After 15 months of storage, LOX activity of RT/C had already reached 11.4 and 5.8 μmin^−1^·g^−1^·FW in HD and Y97 seeds, respectively, which were 6-fold higher than that of LT/V and LT/C.

**Figure 5:**
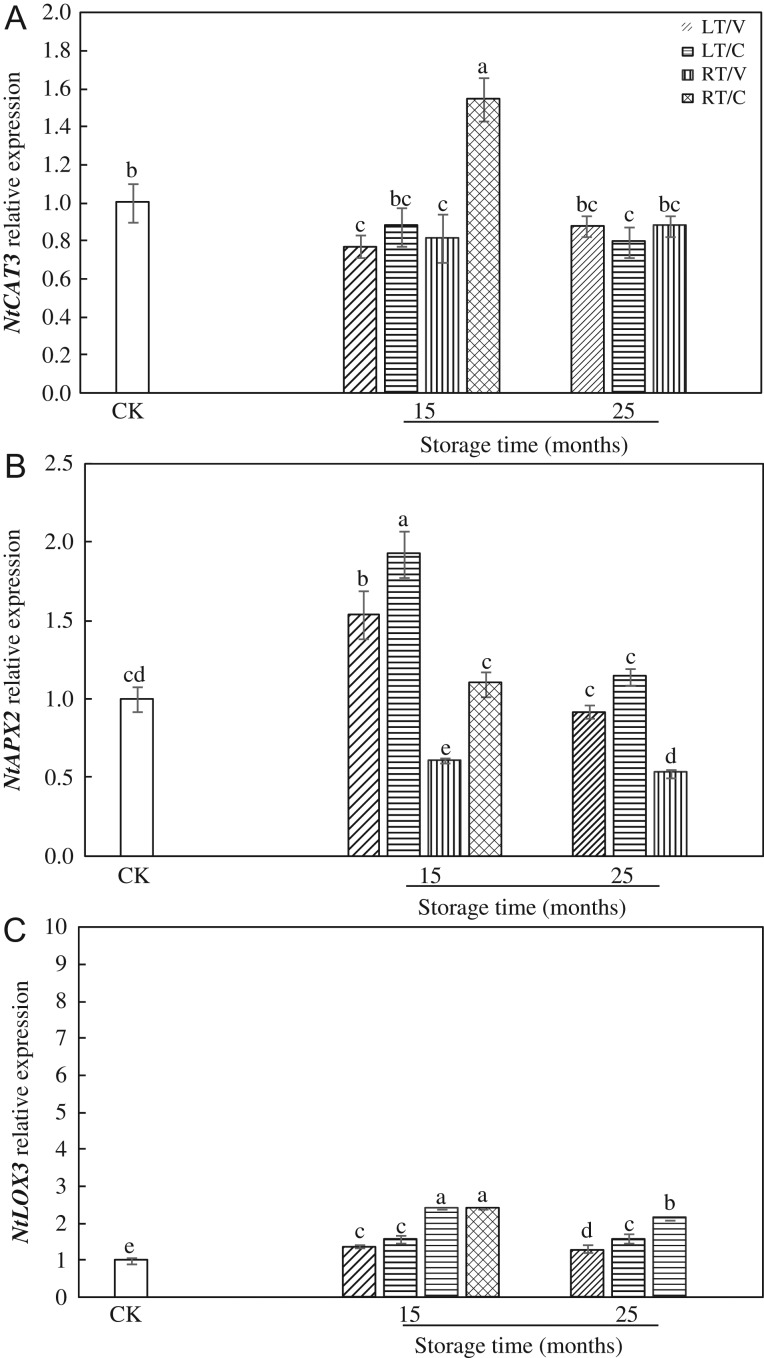
Relative expressions of *NtCAT* (**A**), *NtAPX* (**B**) and *NtLOX3* (**C**) changed in response to different storage conditions in HD seeds during natural aging. HD: Honghua Dajinyuan; Seed samples were collected, respectively, in 0, 15 and 25 months after storage, and four replications for each treatment at each sampling time were used. Different small letter (s) on the top of the bars indicated significant differences (LSD, *α*= 0.05) among treatments at the same storage time. Error bars indicated ± SE of mean (*n* = 4). For additional explanations, see Fig. 1.

The *NtCAT3* expression of RT/C was significantly higher than other treatments after 15-month storage both in HD and Y97 seeds (Fig. 5 A and Supplementary Fig. S6A). The *NtAPX2* expressions in LT/V and LT/C, especially LT/C, were obviously higher than the levels in RT/V and RT/C in HD seeds after 15-month storage (Fig. 5B). While in Y97, all treatments markedly up-regulated the expression of *NtAPX2* except RT/C (Supplementary Fig. S6B).

Four treatments all significantly increased the *NtLOX3* expression levels after 15 months of storage, which in RT/V and RT/C, especially RT/C in Y97 seeds, were obviously higher than those in LT/V and LT/C (Fig. 5C and Supplementary Fig. S6C). In addition, *NtLOX3* expression level of RT/V was significantly higher than LT/V and LT/C after 25-month storage.

#### Regression analysis

The relationship between seed vigour and physiological traits was analysed using regression analysis method (Supplementary Table S3). *X*_1_ (CAT activity), *X*_2_ (APX activity), *X*_3_ (LOX activity), *X*_4_ (H_2_O_2_ content) and *X*_5_ (MDA content) were calculated in five equations. In HD seeds, *R*^2^ values of all equations ranged from 0.163 to 0.982, and the highest was recorded in RT/V with H_2_O_2_ content, followed by LOX activity in LT/V (0.979). It should be noted that only LOX activity had a strong correlation with seed vigour in RT/C, of which *R*^2^ values reached 0.970. However, CAT and APX activities had low correlativity with seed vigour in all equations. The relationship in Y97 seeds was approximately identical with HD seeds. LOX activity, H_2_O_2_ and MDA contents were significantly correlated with seed vigour in LT/V, LT/C and RT/V, LOX activity in RT/C was also significantly correlated with seed vigour in Y97 seeds. Thus, LOX activity was selected as verification object in the following artificial aging.

### Various changes during artificial accelerated aging

#### Seed germination and seedling quality

**Figure 6:**
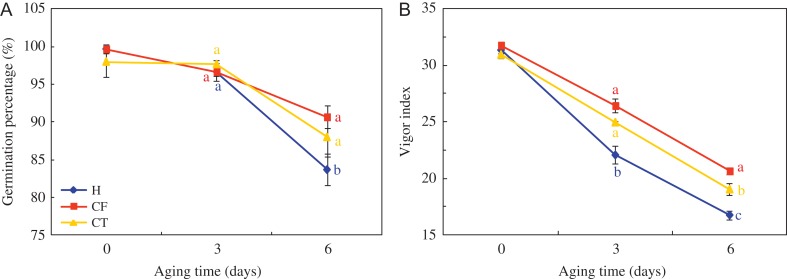
Seed viability and vigour decreased during artificial accelerated aging in HD seeds. Seeds were collected, respectively, on 0, 3 and 6 days after aging. HD: Honghua Dajinyuan. Germination percentage (**A**) was calculated on the 16th day of germination test with four replications for each treatment. Vigour index (**B**) was calculated using the formula: VI = GI×DW, where Gt is the number of new germinated seeds in time Tt, DW is dry weight. Different small letter (s) on the top of the bars indicated significant differences (LSD, *α*= 0.05) among treatments at same storage time. Error bars indicated ± SE of mean (*n* = 4). For additional explanations, see Fig. 1 and Table 2.

VI and GP exhibited a downward trend after artificial aging in HD and Y97 seeds (Fig. 6 and Supplementary Fig. S7). There were no obvious differences among treatments in GP after 3-day aging, while CF and CT had a higher GP than H after 6-day aging. For VI, CF and CT considerably slowed down the reduction rate of VI from 3 to 6 days of aging. In addition, CF recorded the highest GI, SL and DW and the shortest MGT after 3- and 6-day aging in HD (Table 2), followed by CT. For Y97 seeds, GI, SL and DW in CF and CT kept at the same level, and were all higher than that in H pre-treatment (Supplementary Table S4).

**Table 2:** Seed vigour and seedling quality of primed HD seeds during artificial accelerated aging

	Treatments	GE (%)	GI	MGT (d)	SL (cm)	DW (g/50 plants)
0	H*	99.7 ± 0.6a	31.4 ± 0.3ab	3.23 ± 0.06a	1.83 ± 0.08a	0.0078 ± 0.0001a
	CF	99.7 ± 0.6a	31.8 ± 0.8a	3.17 ± 0.16a	1.87 ± 0.05a	0.0075 ± 0.0003a
	CT	98.0 ± 2.1a	30.9 ± 0.4b	3.21 ± 0.13a	2.03 ± 0.04a	0.0073 ± 0.0003a
3	H	96.7 ± 0.6a	22.1 ± 0.3c	4.46 ± 0.01a	1.52 ± 0.18b	0.0042 ± 0.0003b
	CF	96.7 ± 1.2a	26.4 ± 0.6a	3.78 ± 0.04c	1.67 ± 0.05a	0.0057 ± 0.0001a
	CT	97.7 ± 1.5a	25.0 ± 0.3b	4.00 ± 0.02b	1.61 ± 0.16ab	0.0045 ± 0.0008ab
6	H	83.7 ± 2.0b	16.8 ± 0.4c	5.05 ± 0.03a	1.18 ± 0.44b	0.0028 ± 0.0003c
	CF	90.7 ± 0.6a	20.7 ± 0.1a	4.44 ± 0.03c	1.43 ± 0.05a	0.0056 ± 0.0002a
	CT	88.0 ± 2.6a	19.1 ± 0.6b	4.67 ± 0.07b	1.38 ± 0.12ab	0.0048 ± 0.0002b

*Values were mean ± SE (*n* = 4). Different small letter (s) following the values indicated significant difference (LSD, *α* = 0.05) among treatments. Seeds were collected, respectively, on 0, 3 and 6 days after artificial accelerated aging. HD: Honghua Dajinyuan; H: seeds primed with water; CF: seeds primed with caffeic acid; CT: seeds primed with catechuic acid. GE: germination energy, calculated on Day 7; GI: germination index, was measured as GI = ∑ (Gt/Tt); MGT: mean germination time, was calculated as MGT = ∑ (Gt × Tt)/∑Gt, where Gt is the number of new germinated seeds in time Tt; SL: seedling length; DW: seedling dry weight, was weighed directly after drying at 80°C for 24 h.

#### Endogenous H_2_O_2_ and MDA content

**Figure 7:**
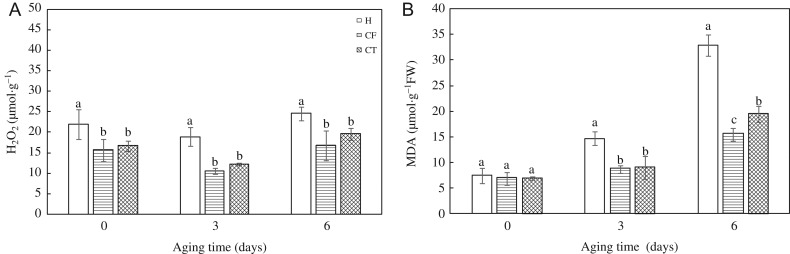
Artificial accelerated aging increased endogenous H_2_O_2_ (**A**) and MDA (**B**) content in HD seeds. Seeds were collected, respectively, on 0, 3 and 6 days after artificial accelerated aging, and four replications for each treatment at each sampling time were used. HD: Honghua Dajinyuan; H_2_O_2_: hydrogen peroxide; MDA: malondialdehyde. Different small letter (s) on the top of the bars indicated significant differences (LSD, *α*= 0.05) among treatments at the same aging time. Error bars indicated ± SE of mean (*n* = 4). For additional explanations, see Table 2.

H_2_O_2_ content in CF and CT was visibly lower than that in H after 3- and 6-days aging in HD and Y97 seeds (Fig. 7A and Supplementary Fig. S8A). In Y97 seeds, H_2_O_2_ content in CF was distinctly lower than CT after 3- and 6-day aging, however, there was no significant difference found in HD seeds. MDA content increased with increased aging time in HD and Y97 seeds, especially 6-day aging (Fig. 7B and Supplementary Fig. S8B). CF and CT significantly decreased MDA content comparing with H, where MDA content in CF was significantly lower than that in CT after 6-day aging.

#### Antioxidant enzymes and LOX activities, and the corresponding gene expression

After 3-day aging, the activities of CAT and APX notably reduced to half of before aging in HD and Y97 seeds. However, there were no significant difference could be found among treatments as well as gene expression after 6-day aging (Supplementary Fig. S9).

**Figure 8:**
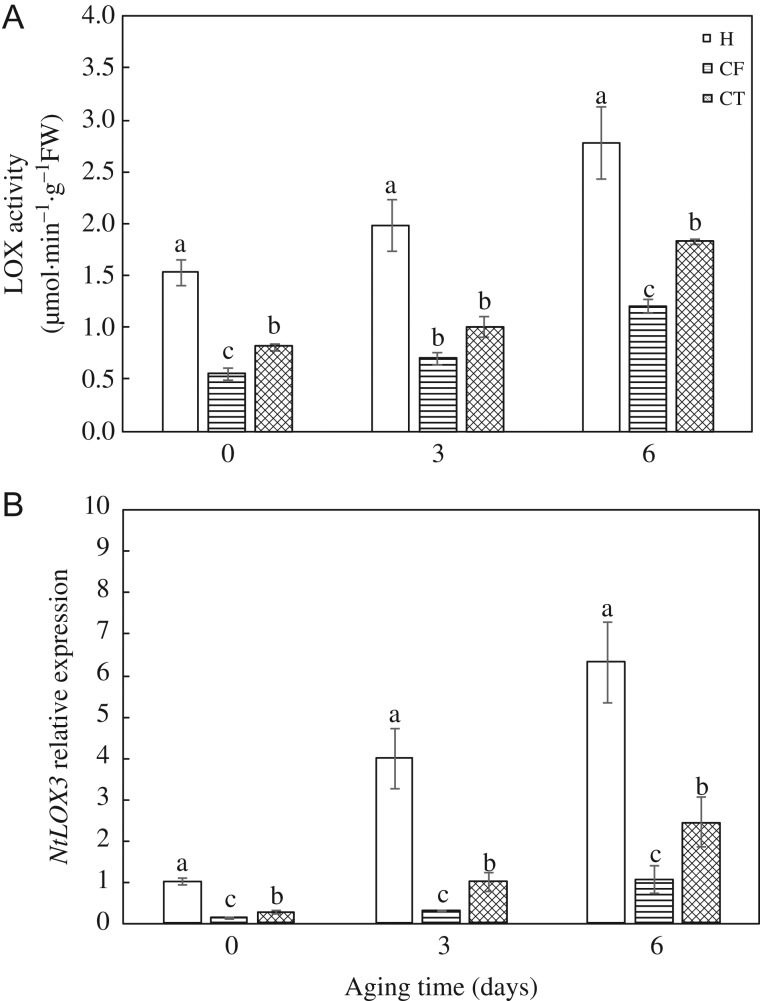
LOX (**A**) activities and *NtLOX3* (**B**) expression increased in response to artificial aging in HD seeds. HD: Honghua Dajinyuan; LOX, lipoxygenases. Seeds were collected, respectively, on 0, 3 and 6 days after artificial accelerated aging, and four replications for each treatment at each sampling time were used. Different small letter (s) on the top of the bars indicated significant differences (LSD, *α*= 0.05) among treatments at the same aging time. Error bars indicated ± SE of mean (*n* = 4). For additional explanations, see Table 2.

LOX activity and gene expression enhanced with increased aging time in HD and Y97 seeds (Fig. 8A and Supplementary S10A), which in CF and CT were markedly lower than those in H. In addition, there were no significant differences in LOX activity between CT and CF after 3-day aging, but after 6-day aging, that of CF was significantly lower than that of CT. The difference in gene expression was early appeared after 3-day aging.

## Discussion

During storage, temperature, moisture and storage time are crucial factors to impact seed germination and vigour (Huang *et al.*, 2003; Martins *et al.*, 2004). Physical, physiological and biochemical property changes during seed storage, including loss of viability, moisture content alterations, organelle membranes damage and lipids decomposition were used to assess the degree of seed deterioration, in which a germination test is the standard method used to evaluate viability of *ex situ* conserved seeds. (Lee *et al.*, 2012; FAO, 2014). In the present study, seed were firstly determined with seed vigour, viability and seedling quality. GE, GI, VI and MGT were used to estimate the speed and uniformity of seed germination, SL and DW coincided with the seedling quality. On the whole, those physiology traits all appeared a downward trend except MGT during natural storage, resulting in a conclusion that aging was associated with loss of seed vigour and viability (Kibinza *et al.*, 2011). By comparison, GI, VI, SL and DW in LT/V were significantly higher and MGT was obviously lower than those in RT/V (Fig. 2, Table 1 and Supplementary Table S2), which suggested that LT had a positive effect on seed storability (Balesevic-Tubic *et al.*, 2010; Pradhan and Badola, 2012). Interestingly, VI sharply decreased after 10 months of storage, and GP subsequently decreased after 15 months especially in RT/C (Fig. 2). The declines in seed vigour occurred 3–6 weeks sooner than in viability during soybean storage (TeKrony *et al.*, 1980). These findings hit that GP was relatively hysteretic in response to deterioration induced by storage, as compared with VI.

Moisture content in RT/C increased sharply to 11.3% and 13.9% (4% of the original moisture content) in HD and Y97 seeds respectively after storage for 20 months (Fig. 1), while RT/V kept a visibly low level during 25-month storage. It indicated packaging design, storage time and temperature jointly affected seed moisture content, which was verified in the analysis of variance for moisture content in Table 3 that the interaction of storage time and storage pattern (packing design and storage temperature) significantly promoted the increase of moisture content as the *P*-value was 0.0001. The reason for the increase in moisture content under RT/C, on the one hand, may relate to the humidity in Yuxi city, which ranged from 52% to 82% during storage (Supplementary Fig. S11). On the other hand, due to the air permeability of cloth bag packing, it made air circulation and moisture transfer possible. When external humidity is higher than the seed moisture content, seed surface and the inner wall of the capillary can absorb water vapour to increase the moisture content of the seed. It also should be pointed out that GP in RT/C was only 30% after 15-month storage and even failed to germinate 5 months later (Fig. 2), whereas GP in RT/V maintained more than 90% at the end of storage. In addition, seed vigour including GE (0), GI (2.0–2.5) and VI (0.009–0.01) in RT/C were also significantly lower than those in RT/V (Table 1, Supplementary Table S1). These results further suggested that packaging design was important for preventing seeds from deterioration during storage. It was in accordance with the analysis of variance for VI (Table 3), and also consistent with the research done by Hopkinson and English (2005) that seed viability in woven bags at ambient temperature was lost in 3 years while barely any change happened in the cold-stored seeds.

**Table 3:** The analysis of variance for moisture content and VI of HD and Y97 seeds

	Variation source	*SS*	*DF*	*MS*	*F*	*P-value*	*F crit*
MC of HD	Storage time*	28.83	5	5.77	1031.68	0.0001	2.62
	Storage pattern	104.70	3	34.90	6245.34	0.0001	3.01
	Storage time × Storage pattern	49.47	15	3.30	590.16	**0.0001**	2.11
	Experimental error	0.13	24	0.01			
MC of Y97	Storage time	53.49	5	10.70	783.18	0.0001	2.62
	Storage pattern	172.05	3	57.35	4198.91	0.0001	3.01
	Storage time × Storage pattern	111.37	15	7.42	543.58	**0.0001**	2.11
	Experimental error	0.33	24	0.01			
	Total variation	337.23	47				
VI of HD	Storage time	0.29	5	0.06	669.77	0.0001	2.41
	Storage pattern	0.08	3	0.03	300.44	0.0001	2.80
	Storage time × Storage pattern	0.04	15	0.00	30.89	**0.0001**	1.88
	Experimental error	0.00	48	0.00			
VI of Y97	Storage time	0.19	5	0.04	174.68	0.0001	2.41
	Storage pattern	0.07	3	0.02	111.13	0.0001	2.80
	Storage time × Storage pattern	0.03	15	0.00	8.99	**0.0001**	1.88
	Experimental error	0.01	48	0.00			

*Time was 0, 5, 10, 15, 20 and 25 months after storage. Pattern was the combination of packing and temperature during storage including LT/V, LT/C, RT/V and RT/C, LT/V: low temperature (18°C) with vacuum bag packing; LT/C: low temperature (18°C) with cloth bag packing; RT/V, room temperature with vacuum bag packing; RT/C: room temperature with cloth bag packing. MC: moisture content; VI: vigour index. The data were analysed by analysis of variance (MANOVA). SS: square sum; DF: degree of freedom; MS: mean square; F: *F*-text. Bold value: the significant level of influence of storage time and storage pattern interaction on the MC or VI.

ROS generated by respiration contributed to aging which in turn lead to the accumulation of H_2_O_2_ in seed cell cytoplasm (Kibinza *et al.*, 2011). Excessive ROS caused lipid peroxidation through LOX, resulting in MDA damaged cell membranes, to further accelerate the seed deterioration (Ahmed *et al.*, 2016; Shimizu *et al.*, 2006; Sharma *et al.*, 2012), which was consistent with our study. H_2_O_2_ and MDA content, and LOX activity kept a similarly upward tendency in RT/C (Figs 3 and 4, Supplementary Figs S3 and S5). The sharpest increase in LOX activity (10 months) was 5 months earlier than that in H_2_O_2_ content (15 months), and the accumulation of MDA content was later than H_2_O_2_ content. Moreover, H_2_O_2_ and MDA content accumulated gradually and constantly in LT/V, LT/C and RT/V during storage (Fig. 3 and Supplementary Fig. S3), while in RT/C, H_2_O_2_ and MDA content rapidly generated and maintained obviously higher levels. And the seed vigour (VI, GE, GI) in RT/C was significantly lower than other treatments from 10-month storage (Table 1 and Supplementary Table S2). Regression analysis indicated that H_2_O_2_ and MDA content had a negative correlation with seed vigour during seed storage (Supplementary Table S3), which indicated excessive accumulation of H_2_O_2_ and MDA was not conductive to the maintenance of seed vigour (Wang *et al.*, 2009; Devaiah *et al.*, 2007). Further evidence was observed in LT/V with the lowest levels of H_2_O_2_ and MDA and the highest seed vigour in HD and Y97 seeds. The probable reason may be that respiration and metabolism in LT (18°C) and vacuum bag packing were slow during storage.

To protect against damage caused by ROS, plants have developed a number of scavenging systems, including antioxidant enzymes. In our study, CAT and APX showed a small fluctuation in response to different storage condition and alternate changed among four treatments during natural aging (Supplementary Fig. S1). Relationship analysis noted that there were no significant associations between CAT, APX and seed vigour (Table 2), although there were studies pointed that seed deterioration is related to a decrease in activities of various peroxide scavenging enzymes (Goel *et al.*, 2003; Xia *et al.*, 2015).

Some other studies reported that the involvement of LOX was determinant in seed storage (Gayen *et al.*, 2014; Huang *et al.*, 2014). The rice variety DawDam, which lacks the *LOX3* gene, exhibited a lower level of unsaturated fatty acid peroxidation and a noticeably decreased level of the stale flavour during seed storage (Suzuki *et al.*, 1999). Xu *et al.* (2015) demonstrated the suppression of *LOX3* expression in rice endosperm increased grain storability. Lima *et al.* (2010) reported that soybean seed of the genotype containing LOXs owned worse seed quality than LOXs loss mutant. During storage, LOX activity in RT/C and RT/V, especially in RT/C, showed a higher level than that in LT/V and LT/C (Fig. 4 and Supplementary Fig. S5), as well as the gene expression (Fig. 5 and Supplementary Fig. S6). These results may indicate that higher temperatures could enhance LOX activity and gene expression. It is worth noting that RT/C contained higher moisture content from 5-month storage and subsequently got a higher LOX activity compared with RT/V. Stored seeds will lose their viability over time to a level at which seed regeneration is required (Walters *et al.*, 2005). Given that LOX is widely existed in plants and closely related to seed vigour (Supplementary Table S3), understanding the relationship between LOX and vigour is essential for germplasm conservation. In this study, the LOX activity in RT/C increased more than 12-fold during 15-month storage, which may demonstrate that tobacco could be a model system to study LOX activity on seed storage.

Except for H_2_O_2_ and MDA content, maybe seed moisture and storage temperature also played an important role in regulating LOX activity. Furthermore, in this study, artificial accelerated aging was used as a method to verify the strong relationship between LOX activity and seed vigour (Supplementary Table S3). The inhibitors of LOX activity, CF and CT, significantly slowed down the reduction of VI, GP and seedling quality in both HD and Y97 seeds during accelerated aging (Supplementary Fig. S3 and Table S4), decreased the content of H_2_O_2_ and MDA (Supplementary Fig. S8), and also decreased LOX activity and down-regulated *NtLOX3* gene expression (Supplementary Fig. S10). On the whole, seed deterioration was alleviated by inhibiting LOX activity.

In conclusion, temperature, storage time and packing design, as well as moisture content of seeds, were considered as crucial factors for maintaining tobacco seed vigour and viability, and extending seed longevity during storage. LT/V was a recommended storage method for tobacco commercial storage or germplasm conservation. Meanwhile, LOX activity played a negative regulatory role in tobacco seed aging because suppression of LOX activity enhanced seed vigour and viability, which could also act as a sensitive signal for predicting seed aging under storage and germplasm conservation. And tobacco seeds may be a model system to study LOX activity on storage based on the reason that LOX activity can change drastically in a short time in tobacco seeds. However, progress on understanding seed aging and longevity has been restricted due to the complexity of the lipid metabolic pathway. Therefore, the potential application of LOX for improving seed storage stability still needed further study.

## Supplementary Material

Supplementary Data
