# Supplementary material for: Suppression of LOX activity enhanced seed vigour and longevity of tobacco (Nicotiana tabacum L.) seeds during storage
Source: Conserv Physiol. 2018 Sep 28;6(1):coy047. doi: 10.1093/conphys/coy047 (PMC6161406; doi:10.1093/conphys/coy047)
Supplement: Supplementary Data [file coy047_revised-suplemantary_data.doc]

**Suppression of LOX activity enhanced seed vigor and longevity of tobacco (*Nicotiana tabacum* L.) seeds during storage**

**Zhan Li^1^, Yue Gao^1^, Cheng Lin^1^, Ronghui Pan^1^, Wenguang Ma^2, 3^, Yunye Zheng^2, 3^, Yajing Guan[[1]](#footnote-2)*, Jin Hu^1^**

1 Seed Science Center, College of Agriculture and Biotechnology, Zhejiang University, Hangzhou 310058, China

2 Yunnan Academy of Tobacco Agricultural Sciences, Yuxi 653100, P.R. China

3 Yuxi Zhongyan Tobacco Seed Company Ltd., Yuxi 653100, P.R. China

**Table S1.** Real-time PCR primers used for genes expression analysis.

| **Gene Name** | **Forward** | **Reverse** |
| --- | --- | --- |
| *NtAPX2* | TGATGTTCCCTTTCACCCTG | TCAGATAGACCCATTTGCTTCAC |
| *NtCAT3* | CCCTAGATGTAACCAAGACCTG | AACAATGGCAGGGCAAAAG |
| *NtLOX3* | TCATTAGCACTGACATCGACC | AGACCAATCCTTTAACACTGCC |
| *NtActin* | CTATTCTCCGCTTTGGACTTGGCA | ACCTGCTGGAAGGTGCTGAGGGAA |

**Table S2.** Seed vigor and seedling quality of Y97 seeds during natural aging.

|  | **Treatments** | **GE (%)** | **GI** | **MGI (d)** | **SL (cm)** | **DW (g/50plants)** |
| --- | --- | --- | --- | --- | --- | --- |
|  | CK^*^ | 96.7±2.5a/a | 30.0±0.8a/a | 3.28±0.01d/c | 1.87±0.08ab/a | 0.0070±0.0006a/a |
| 5 | LT/V | 98.3±1.2 | 28.2±0.9 | 3.58±0.13 | 1.99±0.02 | 0.0054±0.0011 |
|  | LT/C | 99.3±0.6 | 28.8±0.2 | 3.53±0.02 | 1.93±0.11 | 0.0067±0.0005 |
|  | RT/V | 97.7±0.6 | 26.0±0.7 | 3.82±0.11 | 2.04±0.07 | 0.0061±0.0002 |
|  | RT/C | 98.0±1.0 | 24.3±0.3 | 4.08±0.06 | 2.06±0.13 | 0.0056±0.0006 |
| 10 | LT/V | 98.0±2.0 | 27.5±0.8 | 3.65±0.10 | 2.07±0.15 | 0.0053±0.0004 |
|  | LT/C | 98.7±1.2 | 27.4±1.5 | 3.70±0.19 | 2.13±0.06 | 0.0055±0.0003 |
|  | RT/V | 98.0±1.0 | 25.9±1.0 | 3.83±0.10 | 1.62±0.13 | 0.0055±0.0002 |
|  | RT/C | 17.3±5.1 | 11.4±0.4 | 8.28±0.07 | 1.51±0.05 | 0.0036±0.0003 |
| 15 | LT/V | 99.0±1.0a | 27.4±0.6b | 3.67±0.06c | 1.96±0.02a | 0.0053±0.0010b |
|  | LT/C | 99.3±0.6a | 27.9±0.8b | 3.63±0.09c | 1.77±0.15b | 0.0046±0.0009bc |
|  | RT/V | 98.0±1.0a | 25.2±0.3c | 3.91±0.02b | 1.36±0.09c | 0.0035±0.0004c |
|  | RT/C | 0.0 | 2.5±0.5d | 9.00±0.08a | 1.41±0.08c | 0.0043±0.0007bc |
| 20 | LT/V | 97.7±2.1 | 25.9±0.4 | 3.82±0.02 | 1.81±0.12 | 0.0043±0.0004 |
|  | LT/C | 99.7±0.6 | 25.9±0.2 | 3.88±0.01 | 1.80±0.07 | 0.0036±0.0001 |
|  | RT/V | 98.3±1.5 | 21.5±0.5 | 4.67±0.06 | 1.33±0.13 | 0.0040±0.0002 |
|  | RT/C | 0.0 | 0.0 |  |  |  |
| 25 | LT/V | 93.7±1.2a | 24.9±0.3b | 3.81±0.07b | 1.74±0.11a | 0.0041±0.0003b |
|  | LT/C | 94.3±2.5a | 24.8±0.9b | 3.86±0.06b | 1.82±0.05a | 0.0035±0.0001b |
|  | RT/V | 95.0±1.7a | 18.6±0.6c | 5.26±0.07a | 1.25±0.01b | 0.0036±0.0001b |
|  | RT/C | 0.0 | 0.0 |  |  |  |

*Values were mean ± SE (n = 4). Different small letter (s) following the values indicated significant difference (LSD, α=0.05) among treatments. Seed samples were collected respectively in 5, 10, 15, 20 and 25 months after storage. The letters combination such as a/a in the row of CK, indicated the difference (LSD, α=0.05) between CK and treatments after 15 and 25 months of storage, respectively. GE: germination energy, calculated on day 7; GI: germination index, was measured as GI = ∑ (Gt/Tt); MGT: mean germination time, was calculated as MGT =∑ (Gt×Tt) /∑Gt, where Gt is the number of new germinated seeds in time Tt; SL: seedling length; DW: seedling dry weight, was weighed directly after drying at 80°C for 24 h. LT/V: low temperature (18°C) with vacuum bag packing; LT/C: low temperature (18°C) with cloth bag packing; RT/V, room temperature with vacuum bag packing; RT/C: room temperature with cloth bag packing. Different small letter (s) following the values indicated significant difference (LSD, α=0.05) among treatments. Seed storage in RT/C lost germination ability after 20 months, parameters data were no longer concerned.

**Table S3.** The regression analysis between seed vigor and physiological traits in HD and Y97 seeds during natural aging.

| **HD** | | | | **Y97** | | |
| --- | --- | --- | --- | --- | --- | --- |
| ***Y*** | **Regression equation** | ***P*-value** | ***R*^2^** | **Regression equation** | ***P*-value** | ***R*^2^** |
| LT/V | *Y*= – 0.267*X*_1_ + 0.399 | 0.345 | 0.222 | *Y*= 0.081*X*_1_ + 0.047 | 0.795 | 0.015 |
|  | *Y*= 0.029*X*_2_ – 0.267 | 0.088 | 0.558 | *Y*= 0.012*X*_2_ – 0.106 | 0.225 | 0.277 |
|  | *Y*= – 0.124*X*_3_ + 0.241 | 0.001 | 0.979 | *Y*= – 0.069*X*_3_ + 0.194 | 0.004 | 0.840 |
|  | *Y*= – 0.003*X*_4_ + 0.263 | 0.002 | 0.929 | *Y*= – 0.002*X*_4_ + 0.198 | 0.009 | 0.776 |
|  | *Y*= – 0.014*X*_5_ + 0.361 | 0.003 | 0.909 | *Y*= – 0.009*X*_5_ + 0.261 | 0.002 | 0.875 |
| LT/C | *Y*= – 0.407*X*_1_ + 0.530 | 0.172 | 0.407 | *Y*= 0.195*X*_1_ – 0.056 | 0.570 | 0.087 |
|  | *Y*= 0.028*X* _2_ – 0.253 | 0.203 | 0.366 | *Y*= 0.014*X*_2_ – 0.134 | 0.325 | 0.239 |
|  | *Y*= – 0.104*X*_3_ + 0.242 | 0.022 | 0.766 | *Y*= – 0.114*X*_3_ + 0.230 | 0.002 | 0.927 |
|  | *Y*= – 0.002*X*_4_ + 0.001 | 0.003 | 0.908 | *Y*= – 0.003*X*_4_ + 0.261 | 0.001 | 0.944 |
|  | *Y*= – 0.008*X*_5_ + 0.324 | 0.011 | 0.831 | *Y*= – 0.013*X*_5_ + 0.311 | 0.006 | 0.876 |
| RT/V | *Y*= 0.516*X*_1_ – 0.266 | 0.412 | 0.173 | *Y*= – 0.022*X*_1_ + 0.149 | 0.789 | 0.001 |
|  | *Y*= 0.036*X*_2_ – 0.450 | 0.023 | 0.716 | *Y*= 0.014*X*_2_ – 0.169 | 0.282 | 0.279 |
|  | *Y*= – 0.048*X*_3_ + 0.232 | 0.001 | 0.933 | *Y*= – 0.075*X*_3_ + 0.214 | 0.005 | 0.892 |
|  | *Y*= – 0.002*X*_4_ + 0.001 | 0.0001 | 0.981 | *Y*= – 0.002*X*_4_ + 0.218 | 0.006 | 0.881 |
|  | *Y*= – 0.009*X*_5_ + 0.001 | 0.0005 | 0.963 | *Y*= – 0.009*X*_5_ + 0.280 | 0.001 | 0.970 |
| RT/C | *Y*= 0.598*X*_1_ – 0.332 | 0.597 | 0.162 | *Y*= 0.657*X*_1_ – 0.573 | 0.418 | 0.338 |
|  | *Y*= 0.045*X*_2_ – 0.615 | 0.158 | 0.709 | *Y*= 0.022*X*_2_ – 0.354 | 0.600 | 0.160 |
|  | *Y*= – 0.020*X*_3_ + 0.223 | 0.015 | 0.970 | *Y*= – 0.032*X*_3_ + 0.183 | 0.050 | 0.893 |
|  | *Y*= – 0.001*X*_4_ + 0.003 | 0.05 | 0.906 | *Y*= – 0.001*X*_4_ + 0.171 | 0.170 | 0.689 |
|  | *Y*= – 0.006*X*_5_ + 0.002 | 0.06 | 0.88 | *Y*= – 0.004*X*_5_ + 0.205 | 0.096 | 0.816 |

Seeds were collected respectively in 0, 5, 15, 20 and 25 months after storage. HD: Honghua Dajinyuan; Y97: Yunyan97; CAT: catalase; APX: ascorbate peroxidase; LOX: lipoxygenases; H_2_O_2_: hydrogen peroxide; MDA: malondialdehyde. *Y* represented seed vigor, *X*_1_ represent CAT activity in stored seeds, *X*_2_ represent APX activity in stored seeds, *X*_3_ represent LOX activity in stored seeds, *X*_4_ represent H_2_O_2_ content in stored seeds, *X*_5_ represent MDA content in stored seeds. *R*^2^ represented the model *R*-square. For additional explanations, see Table S2.

**Table S4.** Seed vigor and seedling quality of primed Y97 seeds during artificial accelerated aging.

|  | **Treatments** | **GE (%)** | **GI** | **MGI (d)** | **SL (cm)** | **DW (g/50plants)** |
| --- | --- | --- | --- | --- | --- | --- |
| 0 | H^*^ | 98.3±0.6a | 30.6±0.3a | 3.26±0.01a | 2.08±0.14a | 0.0080±0.0012a |
|  | CF | 98.3±2.1a | 30.8±0.5a | 3.25±0.04a | 1.97±0.15a | 0.0071±0.0007a |
|  | CT | 96.3±3.1a | 30.1±0.8a | 3.25±0.02a | 1.95±0.12a | 0.0072±0.0005a |
| 3 | H | 94.0±1.7a | 20.3±0.2c | 4.70±0.10a | 1.29±0.09b | 0.0044±0.0002b |
|  | CF | 97.0±1.0a | 27.8±0.3a | 3.59±0.04c | 1.59±0.05a | 0.0057±0.0001a |
|  | CT | 95.0±2.0a | 26.0±0.2b | 3.76±0.07b | 1.61±0.05a | 0.0052±0.0008ab |
| 6 | H | 81.3±2.5b | 15.5±0.8b | 5.35±0.17a | 1.20±0.03b | 0.0029±0.0003b |
|  | CF | 89.7±1.2a | 22.2±0.2a | 4.05±0.03b | 1.37±0.08a | 0.0055±0.0007a |
|  | CT | 88.7±1.5a | 21.5±0.3a | 4.16±0.02b | 1.46±0.05a | 0.0051±0.0004a |

*Values were mean ± SE (n = 4). Different small letter (s) following the values indicated significant difference (LSD, α=0.05) among treatments. Seeds were collected respectively on 0, 3 and 6 days after artificial accelerated aging. Y97: Yunyan97; H: seeds primed with water; CF: seeds primed with caffeic acid; CT: seeds primed with catechuic acid. GE: germination energy, calculated on day 7; GI: germination index, was measured as GI = ∑ (Gt/Tt); MGT: mean germination time, was calculated as MGT =∑ (Gt×Tt) /∑Gt, where Gt is the number of new germinated seeds in time Tt; SL: seedling length; DW: seedling dry weight, was weighed directly after drying at 80°C for 24 h.

**Fig. S1.** Dynamic changes in moisture content of Y97 seeds during natural aging. Y97: Yunyan97; LT/V: low temperature (18°C) with vacuum bag packing; LT/C: low temperature (18°C) with cloth bag packing; RT/V, room temperature with vacuum bag packing; RT/C: room temperature with cloth bag packing. Vertical bars above mean indicated standard error of 2 replicates of 0.2 g seeds each treatment. Different small letter (s) following the values indicated significant difference (LSD, α=0.05) among treatments. Seed storage in RT/C lost germination ability after 20 months, parameters data were no longer concerned.

**Fig. S2.** Seed viability and vigor decreased as storage continued in Y97 seeds. Seeds were collected respectively in 0, 5, 15, 20 and 25 months after storage. Y97: Yunyan97. Germination percentage (GP, A) was calculated on the 16^th^ day of germination test with four replications for each treatment. Vigor index (VI, B) was calculated using the formula: VI=GI×DW, where Gt is the number of new germinated seeds in time Tt, DW is dry weight. Different small letter (s) on the top of the bars indicated significant differences (LSD, α=0.05) among treatments at same storage time. Error bars indicated ± SE of mean (n = 4). For additional explanations, see Fig.S1.

**Fig. S3**. Seed storage increased endogenous H_2_O_2_ (A) and MDA (B) content in Y97 seeds. Seeds were collected respectively in 0, 5, 10, 15, 20 and 25 months after natural storage, and four replications for each treatment at each sampling time were used. Y97: Yunyan97; H_2_O_2_: hydrogen peroxide; MDA: malondialdehyde. Different small letter (s) on the top of the bars indicated significant differences (LSD, α=0.05) among treatments at the same storage time. Error bars indicated ± SE of mean (n = 4). For additional explanations, see Fig.S1.

**Fig. S4.** Fluctuation of antioxidant enzyme activities under different storage conditions during natural aging in HD and Y97 seeds. HD: Honghua Dajinyuan; Y97: Yunyan97; Catalase (CAT, A in HD, B in Y97) and ascorbate peroxidase (APX, C in HD, D in Y97). Seed samples were collected respectively in 5, 10, 15, 20 and 25 months after storage, and four replications for each treatment at each sampling time were used. Different small letter (s) on the top of the bars indicated significant differences (LSD, α=0.05) among treatments at the same storage time. Error bars indicated ± SE of mean (n = 4). For additional explanations, see Fig.S1.

**Fig. S5.** LOX activity increased accompanied by natural aging in Y97 seeds. Y97: Yunyan97; LOX, lipoxygenases. Seed samples were collected respectively in 0, 5, 10, 15, 20 and 25 months after storage, and four replications for each treatment at each sampling time were used. Different small letter (s) on the top of the bars indicated significant differences (LSD, α=0.05) among treatments at the same storage time. Error bars indicated ± SE of mean (n = 4). For additional explanations, see Fig.S1.

**Fig. S6.** Relative expressions of *NtCAT* (A), *NtAPX* (B) and *NtLOX3* (C) changed in Y97 seed during natural aging. Y97: Yunyan97. Seed samples were collected respectively in 0, 15 and 25 months after storage, and four replications for each treatment at each sampling time were used. Different small letter (s) on the top of the bars indicated significant differences (LSD, α=0.05) among treatments at the same storage time. Error bars indicated ± SE of mean (n = 4). For additional explanations, see Fig.S1.

**Fig. S7.** Seed viability and vigor decreased during artificial accelerated aging in Y97 seeds. Seeds were collected respectively on 0, 3 and 6 days after aging. Y97: Yunyan97. Germination percentage (A) was calculated on the 16^th^ day of germination test with four replications for each treatment. Vigor index (B) was calculated using the formula: VI=GI×DW, where Gt is the number of new germinated seeds in time Tt, DW is dry weight. Different small letter (s) on the top of the bars indicated significant differences (LSD, α=0.05) among treatments at same storage time. Error bars indicated ± SE of mean (n = 4). For additional explanations, see Table S4.

**Fig. S8.** Artificial accelerated aging increased endogenous H_2_O_2_ (A) and MDA (B) content in Y97 seeds. Seeds were collected respectively on 0, 3 and 6 days after artificial accelerated aging, and four replications for each treatment at each sampling time were used. Y97: Yunyan97; H_2_O_2_: hydrogen peroxide; MDA: malondialdehyde. Different small letter (s) on the top of the bars indicated significant differences (LSD, α=0.05) among treatments at the same aging time. Error bars indicated ± SE of mean (n = 4). For additional explanations, see Table S4.

**Fig. S9.** Fluctuation of CAT (A, B) and APX (C, D) activities and relative expressions of *NtCAT3* (E, F), *NtAPX2* (G, H) during artificial aging. A, C, E and G were measured in Honghua Dajinyuzn (HD) seeds; B, D, F and H were in Yunyan97 (Y97) seeds. Seeds were collected respectively on 0, 3 and 6 days after artificial accelerated aging, and four replications for each treatment at each sampling time were used. Different small letter (s) on the top of the bars indicated significant differences (LSD, α=0.05) among treatments at the same storage time. Error bars indicated ± SE of mean (n = 4). For additional explanations, see Table S4.

**Fig. S10.** LOX (A) activities and *NtLOX3* (B) expression increased in response to artificial aging in Y97seeds. Y97: Yunyan97; Seeds were collected respectively on 0, 3 and 6 days after artificial accelerated aging, and four replications for each treatment at each sampling time were used. Different small letter (s) on the top of the bars indicated significant differences (LSD, α=0.05) among treatments at the same aging time. Error bars indicated ± SE of mean (n = 4). For additional explanations, see Table S4.

**Fig. S11.** Temperature and humidity changes from June 2015 to June 2017 in Yuxi city, Yunnan Province (102.52E, 24.35N), China.

1. ^*^Corresponding author. Tel.: +86 57188982318; fax: +86 57188982318; E-mail address: vcguan@zju.edu.cn [↑](#footnote-ref-2)
